# Acute and Chronic Ethanol Effects during Adolescence on Neuroimmune Responses: Consequences and Potential Pharmacologic Interventions

**DOI:** 10.3390/cells12101423

**Published:** 2023-05-18

**Authors:** Kala N. Nwachukwu, Hassan E. Mohammed, DaQuan R. Mebane, Andrew W. Barber, H. Scott Swartzwelder, S. Alex Marshall

**Affiliations:** 1 Department of Biological & Biomedical Sciences, North Carolina Central University, Durham, NC 27707, USA; 2 Integrated Biosciences PhD Program, North Carolina Central University, Durham, NC 27707, USA; 3 Department of Psychiatry and Behavioral Sciences, Duke University Medical Center, Durham, NC 27708, USA

**Keywords:** adolescent alcohol use, microglia, astrocytes, cytokines, toll-like receptors, neuroimmune system

## Abstract

Heavy ethanol consumption during adolescence has been linked to neuroimmune response dysregulation and cognitive deficits in the developing adolescent brain. During adolescence, the brain is particularly susceptible to the pharmacological effects of ethanol that are induced by acute and chronic bouts of exposure. Numerous preclinical rodent model studies have used different ethanol administration techniques, such as intragastric gavage, self-administration, vapor, intraperitoneal, and free access, and while most models indicated proinflammatory neuroimmune responses in the adolescent brain, there are various factors that appear to influence this observation. This review synthesizes the most recent findings of the effects of adolescent alcohol use on toll-like receptors, cytokines, and chemokines, as well as the activation of astrocytes and microglia with an emphasis on differences associated with the duration of ethanol exposure (acute vs. chronic), the amount of exposure (e.g., dose or blood ethanol concentrations), sex differences, and the timing of the neuroimmune observation (immediate vs. persistent). Finally, this review discusses new therapeutics and interventions that may ameliorate the dysregulation of neuroimmune maladaptations after ethanol exposure.

## 1. Introduction

Binge drinking among adolescents is a significant problem within the United States. Adolescence is often considered the time period from age ten to twenty-four years old in human development, but the onset and end of adolescence vary by sex. Moreover, even within adolescence, there are differences between brain development in early versus late adolescence. Puberty is a critical window within adolescence marked by various physiological changes including sexual maturation. However, as one reaches puberty, risk-taking behaviors increase due to the constant neurological events and developments that shift neuronal circuits [1]. Some of these behaviors include binge drinking, smoking, and risky sexual behavior [2,3,4]. It has been shown that adolescents are more likely to explore and try novelties, including marijuana and alcohol, at high levels. In the United States, approximately 12% or 4.2 million underage individuals (12–20) reported binge drinking at least once in the past month, but the prevalence of binge drinking increases to over 35% between the legal drinking age, 21, and the clinical end of adolescence at 25 [5]. Individuals who began drinking before the age of 15 are also 4-times more likely to develop an Alcohol Use Disorder (AUD) at some point in their lives. One hypothesis for this increased susceptibility for an AUD is that neuroplastic maladaptations that occur during adolescence have long-term consequences that impact alcohol pharmacology and related behaviors.

During adolescence, brain development is especially susceptible to insults which have persisting neurological effects. This is particularly concerning given that the human brain continuously develops beyond the legal drinking age in many countries, including the US, and that societal attitudes, easy accessibility to alcohol, and environmental factors have normalized binge drinking among adolescents [6]. There are various aspects of the central nervous system (CNS) that have been studied to determine the maladaptive effects and factors that modify the consequences of excessive alcohol use among adolescents that have been reviewed, including effects on neurogenesis, sex as a biological variable, anxiety-like behavior, learning and memory, structural and cognitive deficits, cholinergic function, and epigenetic modifications. Although it is important to recognize that many of these systems are interconnected, this review will focus on the neuroimmune system.

Neuroinflammation is a complex biological response to invading pathogens and tissue that is initiated to protect the CNS from damage, but when the system is perpetually stimulated, it can exacerbate damage. Clinical studies have indicated that there are distinct changes to various aspects of the neuroimmune system after a lifetime of an AUD, suggestive of long-term neuroinflammatory responses, including changes in microglial activation, increased expression of receptors that mediate the inflammatory response, and changes in central and peripheral cytokine levels [7,8]. However, the complexity of the neuroimmune system has led to a variety of studies looking at nuances that may change the neuroimmune response, including periods of abstinence, the chronicity of the exposure, age, and the dose. Moreover, these changes in mediators of the neuroimmune response have been linked to polymorphisms or peripheral biomarkers, suggesting a strong tie between the susceptibility to develop an AUD and the neuroimmune system.

**Figure 1:**
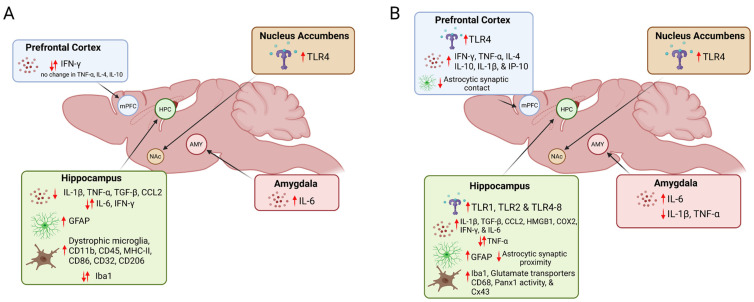
Summative figure of acute (**A**) and chronic (**B**) effects of adolescent alcohol exposure on neuroimmune responses. Arrows indicate the direction of the changes in responses in microglia, astrocytes, cytokines, and TLRs. When studies indicated conflicting results, a bidirectional arrow is included. Created with BioRender.com.

Considering the reciprocal, reinforcing relationship between the neuroimmune system and alcohol consumption, this review seeks to provide an update on the newest literature (within the last five years) comparing acute and chronic adolescent alcohol use models and how they impact the neuroimmune system, as well as describing the current efforts to identify new drugs and treatments to combat these neuroimmunological maladaptations. More specifically, we will highlight the effects of excessive adolescent alcohol use on toll-like receptors, cytokines and chemokines, microglia, and astrocytes. Here, we define acute alcohol exposure as short-term bouts of binge-like ethanol concentrations within 1–4 days and chronic alcohol exposure as long-term consumption of binge-like concentration over 10+ days in relationship to rodent development. Moreover, only studies where the alcohol exposure was specifically within the adolescent window are included to highlight the vulnerability of the CNS during this critical period. Within our analysis, this review seeks to highlight characteristics that influence the responses, including sex, duration/dose of alcohol, regional, and immediate versus persistent neuroimmune responses. Figure 1 shows the findings from acute (Figure 1A) and chronic (Figure 1B) ethanol exposure in the context of regional differences, but the tables throughout provide additional context to consider the aforementioned caveats’ (e.g., sex) influences on these generalized neuroimmune responses to adolescent alcohol use.

## 2. Acute and Chronic Effects of Adolescent Binge Drinking on Toll-like Receptors

Toll-Like Receptors (TLRs) are a family of receptors that help to mediate both the peripheral immune response as well as the neuroimmune system. They are expressed in the CNS on glia (microglia, astrocytes, and oligodendrocytes) as well as on neurons [9], and as previously stated, clinical studies indicate an increase in TLRs in the CNS in postmortem samples of individuals with an AUD. In vitro studies and AUD models in adults agree with the critical role of TLRs, indicating that alcohol agonizes TLR systems through increases in expression or indirectly through upregulation of inflammatory mediators that are TLR agonists [10,11,12]. TLR4, in particular, has been repeatedly studied for its role in AUDs and other substance use disorders [13]. TLR4 is a part of the pattern recognition receptors (PRRs) that detects pathogen-associated molecular patterns (PAMPs), enabling it to be a key receptor that stimuli and injury use to induce a proinflammatory response. Although there are not a lot of recent studies that have focused on the acute effects of adolescent binge-like alcohol consumption on TLRs, Jacobsen and colleagues observed increased TLR4 mRNA levels in the nucleus accumbens of adolescent mice who received just four doses of ethanol that persisted into adulthood [14]. Importantly, there did appear to be a dose relationship with animals over the binge limit exhibiting an increase in TLR4 expression [14]. It is possible that multiple days of acute ethanol are necessary to elicit changes to the TLRs, as two doses on a single day decreased TLR4 levels in the cornu ammonis 1 (CA1) of the hippocampus during late adolescence [15]. This study emphasizes that even small bouts of binge drinking during adolescence can have long-term effects, dependent upon the duration of exposure, developmental period, and region studied.

There are more studies that highlight the effects of chronic adolescent ethanol exposure on TLRs. For example, following the adolescent intermittent ethanol (AIE) exposure model, an upregulation of TLR1, TLR2, TLR4, TLR5, TLR6, TLR7, and TLR8 mRNA was observed in the adult hippocampus of male rats [16]. However, changes to TLRs were not universal in their study as no effect of AIE was observed in TLR3 or TLR9 mRNA. The lack of a TLR3 response to ethanol contrasts with previous studies that indicate that TLR3 is upregulated in the PFC and cerebellum [17,18]. Similar findings of an increase in TLR4 protein following twenty-one days of free access to 10% ethanol (*v*/*v*) were also observed in male rats. Moreover, new evidence has supported that sex may be a biological factor that plays a role in the long-term alcohol-induced effects on TLR4 expression. Using a persisting self-administration model in early adolescent male and female rats, Silva-Gotay and colleagues observed regional- and sex-specific effects of binge drinking on the TLR4 gene. Persistent alcohol binge drinking caused a significant increase in TLR4 gene expression in the medial prefrontal cortex (mPFC), specifically in male but not female rats, but they detected a significant increase in TLR4 mRNA expression in the hippocampus of both sexes [19]. Studies on the long-term impact of adolescent chronic ethanol exposure on TLRs seem more congruent than those for acute exposure, but there are still potential influential factors such as sex that require exploration. Furthermore, the role of adolescent alcohol-induced increases in TLR4 in maladaptive behaviors such as excessive consumption and in perpetuating cellular damage suggest that careful attention should be paid to understanding the influence of alcohol on the entire family of TLRs. A summary of the most recent findings is available in Table 1.

## 3. Acute and Chronic Effects of Adolescent Binge Drinking on Cytokines and Chemokines

Cytokines and chemokines are small proteins released as a part of the neuroimmune response and act as both paracrine and autocrine signals that can enhance (proinflammatory) or dampen (anti-inflammatory) the neuroinflammatory response. In the CNS, cytokines and chemokines (as well as their receptors) are not only expressed by neuroimmune cells but are also expressed by neurons and endothelial cells. Both clinical studies of peripheral cytokines and postmortem studies of the CNS have shown that alcohol alters cytokines [8,21]. The dynamic expression of cytokines suggests that there are many potential differences between acute and chronic exposures to ethanol. Following 4 days of adolescent ethanol exposure, Peng and Nixon observed a downregulation of both pro- and anti-inflammatory cytokine mRNA in isolated microglia, specifically a decrease in interleukin-1 beta (IL-1β), interleukin-6 (IL-6), transforming growth factor beta (TGF-β), and tumor necrosis factor alpha (TNF-α), as well as the chemokine C-C motif chemokine ligand 2 (CCL2) in the hippocampus and entorhinal cortex [22]. This finding agrees with a recent mouse study that observed decreases in peripheral cytokines after acute ethanol exposure [23]. Others have seen increases in IL-6 and other immune-regulating proteins after more acute exposure [24]. IL-6 is an interesting cytokine because it has been characterized as both pro- and anti-inflammatory depending on the environment [25], so its role after acute ethanol exposure is still fairly ambiguous. Finally, in an acute adolescent ethanol exposure model by Bellozi et al., no changes in the expression of cytokines TNF-α, interleukin-4 (IL-4), and interleukin-10 (IL-10) in the prefrontal cortex and hippocampus were observed after two distinct doses, but there was a dose response change in interferon gamma (IFN-γ) in the PFC after ethanol exposure [26]. The discrepancy in findings between these studies may be associated with the timing of the observations. The animals in the Majchrowicz and acute mouse model were euthanized while intoxicated, whereas the Bellozi study waited 2 days after ethanol exposure [26]. Previous studies have highlighted the important role of timing (e.g., intoxication, withdrawal, and abstinence) in understanding the impact of ethanol on cytokines.

The AIE chronic ethanol exposure paradigm has revealed that adolescent ethanol causes a persistent increase in various inflammatory modulators, including chemokine CCL2, cyclooxygenase-2 (COX2), high-mobility group box 1 (HMGB1) as well as cytokines TNF-α, IL-6, and IL-1β in the hippocampus into adulthood. However, these findings are not universal as some have reported a decrease in TNF-α and no change in IL-1β in similar AIE studies, but other chronic adolescent models (i.p. injections and free access 10% ethanol) have shown an increase in IL-1β, TNF-α, and IL-6 in the hippocampus [20,27]. Additionally, studies have observed an increase in IL-6 and C-X3-X Motif Ligand 1 (CX3CL1) in the striatum in adulthood after 3 weeks of ethanol abstinence [28]. Interestingly, cytokines levels after ethanol exposure were exacerbated by a high-fat diet [28]. Additionally, it has been reported that ethanol-induced cytokine expression can vary between sexes [29]. For example, we observed a significant increase in cytokines TNF-α and IL-1β in the adult hippocampus of male rats but not in female rats [30]. The persisting effects of chronic ethanol exposure seem to be more impactful to proinflammatory molecules as there were no changes in the expression of anti-inflammatory cytokines IL-4 or IL-10 in the hippocampus, prefrontal cortex, or whole brain. Overall, the effects of ethanol exposure on pro-inflammatory cytokines and chemokines are complex, with distinct regional specificity that changes dynamically based on whether the ethanol exposure is acute or chronic. See Table 2 for specifics about various regions and specific cytokines.

## 4. Acute and Chronic Effects of Adolescent Binge Drinking on Microglia

Microglia act as the first responders within the neuroimmune system, recruiting other microglia, astrocytes, and even peripheral immune cells through the release of chemokines and cytokines [32,33]. Research has shown the involvement of microglia in the neuroimmune response succeeding dependent and non-dependent binge drinking [34,35]. Moreover, we know that early life events that stimulate the neuroimmune system can result in primed microglia [36,37,38]. Microglial priming is a phenomenon by which microglia have an enhanced response to secondary immunomodulators due to a persisting phenotypic shift (e.g., expression of receptors, cluster differentiation factors, etc.) after the first noxious stimuli. While the full effects of alcohol use on the microglial response are still under investigation, there is evidence that ethanol’s influence on microglia results in a primed state, including after adolescent exposure. Considering the potential long-term effects of ethanol on microglia, it is important to understand the effects of excessive ethanol on microglia during the vulnerable phase of adolescence. After acute ethanol consumption, a substantial reduction in microglia in the hippocampus, perirhinal, and entorhinal cortices was observed in adolescent rats following a 2-day or 4-day binge [39]. The reduction in the number of microglia was concurrent with dystrophic morphology, indicative of a potential deficit during intoxication. Similar experiments suggest that the dystrophic microglia may be a subset as there does appear to be an increase in overall microglial activation according to ionized calcium-binding adaptor molecule 1 (Iba1) morphological assessments [40]. An analysis of the molecular profile of microglia after this Majchrowicz model indicates that both M1 and M2 microglia are present, suggesting that the microglial phenotypic response after adolescents consume alcohol is complex, representing the continuum of microglial pro- and anti-inflammatory responses [22]. However, a study using a model with lower blood ethanol concentrations (BECs) (~200 mg/dL) reported no changes in microglia activation or microglia TLR4 expression following a two-day adolescent alcohol binge exposure [41]. The discrepancy in these reports suggests that there may be a threshold of ethanol concentrations that alter the microglial profile.

Studies of the chronic effects of adolescent ethanol misuse on microglia in rats, conducted in our lab and others using the AIE model, have indicated a significant increase in Iba1 immunoreactivity in the hippocampus [42,43]. However, there were some discrepancies in these studies concerning sex, as one study reports Iba1 increases in males while we have shown that Iba1 increases were mainly driven by female rats [43]. In similar studies on male mice, AIE caused an increase in cluster of differentiation molecule 68 (CD68), indicative of phagocytic activity in microglia in other brain regions, including the dorsal and medial raphe nuclei as well as the dorsal horn or increased Iba1 density in the prefrontal cortex, but the findings are not universal. One study, using a mouse AIE model, observed a loss of hippocampal microglia after AIE, but the animals had also experienced stressful behaviors, which may have been a confounding factor in the microglial expression [44,45]. Moreover, in the same study, re-exposure to ethanol resulted in a bifurcated response with evidence of microglial proliferation concurrent with apoptotic-positive microglia [44]. These findings are reminiscent of the acute exposure and priming effects that have previously been seen in adult AUD and fetal alcohol spectrum disorder models, suggesting that the long-term effects of ethanol from adolescent exposure reprogram microglia to respond to future alcohol exposure. The microglial effects are not paradigm specific, with self-administration showing long-term effects of increased Iba1 in the medial prefrontal cortex and hippocampus [19]. Likewise, ethanol vapor exposure in adolescence showed that immediately after exposure, there was an increase in activated microglia in the amygdala, frontal cortex, hippocampus, and substantia nigra, with mixed results concerning the long-term effects [46]. Across species and models, it seems clear that microglia retain plastic changes following chronic adolescent alcohol exposure that alter the microenvironment, but there remain discrepancies concerning the regional specificity, influence of sex, as well as the functional implications of these long-term changes (see Table 3).

## 5. Acute and Chronic Effects of Adolescent Binge Drinking on Astrocytes

Astrocytes are known to play a critical role in modulating glutamate homeostasis and are integral to blood–brain barrier maintenance, promoting healthy synaptic connectivity, and, importantly for this review, astrocytes can modulate neuroimmune responses [47,48]. Research from the mid-1980s indicated that binge drinking can alter astrocytes, but their functional consequence and potential as a therapeutic target are currently under investigation [49,50,51]. Using a binge-like ethanol intake model to assess the short-term effects of binge ethanol consumption on astrocytes, female rats underwent intragastric administration of ethanol for 1 or 4 cycles during early adolescence in a 3-days-on/4-days-off paradigm [52]. Increases in glial fibrillary acidic protein (GFAP), a well-known marker for astrocytes, were measured in the hippocampus, through Western blot analysis after both one and four cycles, suggesting astrocyte activation immediately after acute and chronic ethanol exposure [52]. Interestingly, this experiment was performed in only female rats, due to growing evidence that female rodents have a more robust neuroinflammatory and neuronal injury response to ethanol-induced brain trauma than male rodents [53]. Conversely, others have shown no increases in GFAP-positive cells following four doses of ethanol in rats, but perceived differences in these observations may be due to the outcome measured. Activated astrocytes may change their morphology, resulting in more GFAP expression, but this does not mean that there has been a proliferative event resulting in more astrocytes. The ways that astrocyte activation can be measured and classified should be carefully considered when making conclusions about the effects of ethanol on astrocytes [54].

In chronic ethanol exposure studies, there appears to be more consensus. For example, recent work in our lab showed a significant increase in immunoreactivity and GFAP-positive cells in the dentate gyrus, CA1, and CA2/3 hippocampal subregions of AIE exposed male and female rats [43]. This was one of the first studies to show that these alcohol-induced astrocytic effects from adolescence were in females, but surprisingly, there was not an exacerbated effect in females, which has been seen in adult models of AUDs [55,56]. Similar increases in GFAP expression were measured in the hippocampus via ELISA as well as Western blots in the hypothalamus and the hippocampus but not in the prefrontal cortex.

Recent studies have started to examine the molecular changes in astrocytes after adolescent alcohol exposure as well as how they may interact with other neuroimmune events. For example, the effects of non-dependent adolescent binge drinking in the “drinking in the dark” (DID) paradigm and mild traumatic brain injury (mTBI) were examined by Mira and colleagues. This study suggested that the increases in astrocytic reactivity in the hippocampus after DID contribute to worse outcomes after mTBI, but the direct functional relationship between changes in astrocyte morphology/proliferation caused by alcohol on the mTBI remains unknown. However, more investigations have been conducted to look at changes in receptors and transporters in astrocytes after adolescent alcohol exposure that may explain exacerbated neurodegenerative responses. For example, in a study by Bonilla-Del Riotao et al., they found that adolescent mice exposed to chronic doses of ethanol had a significant decline in CB_1_ receptors in astrocytic processes in comparison to their controls [57]. It is hypothesized that this decrease in astrocytic CB_1_ receptors may diminish astrocyte responses to endogenous cannabinoids and impact their role in neuroprotection in the hippocampus.

Changes in astrocyte receptors are not the only way that adolescent alcohol exposure may alter the neural microenvironment. For example, recent AIE studies have shown that AIE reduces astrocyte–synaptic proximity in adulthood in the mPFC [58] and hippocampus [59], specifically with glutamatergic synaptic puncta. It appears that the astrocyte–synaptic relationship deficit requires a period of abstinence before the maladaptation is visible as no effects were seen when examining PSD-95 and GFAP proximity immediately after AIE [60]. However, when examined in adulthood, in the same study, there were deficits in the puncta to astrocyte ratio. Moreover, glutamatergic signaling in these regions can also be modified through astrocytic control of synaptic glutamate concentrations via glutamate transporters. AIE upregulated the astrocytic glutamate transporter GLT-1 in the dorsal hippocampus of male and female rats, while also decreasing astrocyte–neuronal synaptic proximity. Some sex-specific differences were reported as there was an increase in postsynaptic density 95 expression in female rats only, reducing NMDA receptor subunit 2A expression, EphA4, and GLAST in male rats specifically [61].

One mechanism by which astrocytes may alter both neuroimmune signaling and neurotransmission is hemichannels and pannexons. These channels are thought to allow for gliotransmission of ATP, and glutamate functionality in astrocyte-mediated gliotransmission in hippocampal plasticity has also begun to be explored [62]. Chronically ethanol exposed rats were assessed for modifications in the activity of astrocyte hemichannels and pannexons in the hippocampus. The results indicated that adolescent ethanol exposure increased the opening of connexin 43 hemichannels and pannexin-1 channels in astrocytes, which was correlated with an upregulation of proinflammatory cytokines [27]. Together, these recent findings reinforce previous work, indicating that astrocytes can develop maladaptations from adolescent alcohol misuse, but they make a few key distinctions regarding sex as a biological variable’s contributions to alcohol responses as well starting to elucidate the molecular changes that are specific to astrocytes after high levels of adolescent alcohol consumption. The dynamic changes in astrocytes in AUDs remain a topic of interest that represents a potential avenue for therapy.

## 6. Alcohol Use Interventions and Drug Treatments

There are currently three approved medications for the treatment of AUDs: disulfiram, naltrexone, and acamprosate. None of these drugs directly target the neuroimmune system, but there is some evidence that suggests altering the neuroimmune response may afford protection from alcohol-induced neurodegeneration as well as influence alcohol-related behaviors including consumption [63,64,65,66]. In fact, naltrexone itself has some anti-inflammatory properties that have been shown to protect from some of the negative effects of alcohol [14,67]. Here, we also highlight recent work in adolescent alcohol studies that used novel drug approaches that also have the potential to act as neuroimmune modulators, including anticonvulsant drugs, acetylcholinesterase inhibitors, opioid antagonists, lipid-lowering agents, classic anti-inflammatory drugs, and even regular exercise. Neuroimmunomodulation in adult AUD models has been shown to ameliorate alcohol-related brain damage and associated behaviors as well as even reduce consumption [63,68].

### 6.1. Anticonvulsant Drugs

With new emerging studies on the acute and chronic impacts of adolescent binge drinking and its impact on the neuroimmune system, researchers have made promising progression towards interventions and drug treatments to combat these maladaptations. The anticonvulsant drug, gabapentin (Neurontin), is currently a prescribed drug for the treatment of seizures and nerve pain caused by shingles due to its modulation of GABA synthesis and actions on voltage-gated calcium channels, but gabapentin may owe some of its therapeutic effects to its ability to diminish neuroimmune responses by reducing cytokine release and glial activation [69,70]. Gabapentin has been proposed as a potential therapy for AUDs, and clinical trials have shown some efficacy of gabapentin for AUD patients with alcohol-withdrawal symptoms [71,72]. Gabapentin has been shown to reverse long-term changes associated with AIE-induced increases in the NMDA-mediated current in the hippocampus [73]. Importantly, a follow-up study was conducted using a sub-chronic injection treatment of gabapentin, where Healey and colleagues demonstrated that gabapentin also altered astrocytes [58]. More specifically, they observed a reversal of the AIE-induced reduction in astrocyte–synapse interactions in the medial pre-frontal cortex of rats [58]. The mechanism of this gabapentin effect is still of interest but, given its effects on both astrocytes and synaptic transmission, it is reasonable to conclude that it is likely multi-factorial and likely includes some neuroimmune modulation.

### 6.2. Acetylcholinesterase Inhibitors

Current pharmacological treatments for Alzheimer’s and dementia caused by Alzheimer’s are acetylcholinesterase (AChE) inhibitors donepezil and galantamine. In more recent years, AChE inhibitors have been investigated for potential roles in alleviating increased neuroinflammatory responses, restoring dendritic integrity, and increasing neurogenic markers following adolescent alcohol misuse. In an AIE exposure model, Mulholland et al. found that ethanol led to a significant decrease in dendritic spine density and increased mRNA levels of the *Fmr1* gene in the hippocampus into adulthood [74]. These effects were reversed after donepezil administration, reverting dendritic spine morphology back to normal and reducing the upregulated mRNA expression levels of Fmr1 in the adult hippocampus of rats [74]. A follow-up study to assess the efficacy of donepezil in neuroimmune responses, neurogenesis, and neurodegeneration following AIE was also carried out. Donepezil was able to increase the immunoreactivity of the neurogenic marker doublecortin (DCX) back to normal levels, while also decreasing neuronal death marker caspase3 into adulthood. Importantly, neuroinflammatory markers, such as HMGB1, RAGE (Receptor for Advanced Glycation End Products), and pNFκB p65 (Nuclear Factor-κB), were also decreased in adulthood following donepezil administration. Likewise, galantamine has been shown to have similar reversing effects on neuroimmune markers following AIE. A recent study showed that galantamine administration during AIE or following AIE was able to block or reverse the AIE-induced expression of the proinflammatory receptors TLR4 and RAGE, HMGB1, and the transcription activation marker pNFκB p65. Galantamine was also able to prevent and treat a loss of neurogenic marker DCX immunoreactivity, while also reversing proinflammatory markers, chemokine (C-C motif) ligand 2 (CCL2), cyclooxygenase-2 (COX-2), and high-mobility group box 1 (HMGB1) protein [75]. This reversal in neuroimmune signaling may underlie galantamine’s ability to recover the persistent loss of cholinergic neurons in AIE-exposed adult rats and represent a novel treatment for AUDs.

### 6.3. Opioid Antagonist

The mu opioid antagonist naltrexone is FDA-approved to treat AUDs, but other opioid antagonists have also been used and designed to treat AUDs, including nalmefene and Nalmefene, and other opioid antagonists have also been identified as TLR4 antagonists, which have been suggested to alleviate proinflammatory signaling induced by alcohol at these receptors. In a previous study, Montesinos et al. showed that TLR4 was critical to AIE induction of neuroimmune responses, myelin damage, and behavioral dysfunctions, but nalmefene averted the upregulation of neuroimmune responses, in particular cytokines (IL-1β, IL-17A, TNF-α), chemokines (MCP-1, MIP-1, KC), and proinflammatory modulators (iNOS, COX-2), in the prefrontal cortex and nucleus accumbens, while diminishing ethanol-induced preference and consumption [76]. These findings were not observed when nalmefene was administered to TLR4 knockout mice [76]. Furthermore, using in vitro primary cultured astrocytes from female C57BL/6 mice and TLR4 knockout mice, their findings indicated that nalmefene can perturb TLR4 activation succeeding ethanol induction [76]. Additionally, nalmefene pre-treatment in a chronic alcohol exposure rat model system reduced TSPO in a PET imaging study, showing a significant decrease in alcohol-induced neuroimmune responses in all brain regions [77]. Similar findings have been observed with naltrexone. More specifically, naltrexone pretreatment blocked an increase in TLR4 genes in adulthood [14]. Exploiting the duality of opioid antagonists and their role in opioid signaling as well TLRs may lead to changes in alcohol craving as well as the neuroimmunological maladaptations caused by early alcohol use.

### 6.4. Lipid-Lowering Agents

More recently, studies have begun to explore the use of fenofibrate, a peroxisome proliferator-activated receptor α (PPARα) agonist, for AUD treatment, although it is currently prescribed to reduce cholesterol [78,79]. Astrocytes and microglia both express PPARα, and their activation can be inhibited by PPARα agonists. Utilizing an AIE model, Villavicencio-Tejo et al. tested whether fenofibrate would alleviate AIE-induced neuroinflammation. They found that oral gavage of fenofibrate during the abstinence period significantly ameliorated alcohol-induced GFAP increases in the hypothalamus and hippocampus as well other markers of neuroinflammation [80]. In addition to its neuroimmune effect, fenofibrate was able to reverse alcohol-induced decreases in GLT-1 in the prefrontal cortex and hippocampus back to baseline levels. These results suggest that fenofibrate may be an effective drug for diminishing alcohol-induced astrogliosis and modulate astrocytic roles in neuroinflammation and glutamate regulation. Likewise, γ-oryzanol, a group of chemicals found in rice bran oil, is purported to have some cholesterol-lowering potential, antioxidant properties, and anti-inflammatory capacity [81,82,83]. In a recent study, γ-oryzanol administration after adolescent alcohol exposure led to a reduction in mRNA levels of Il-1β in AIE male mice and reduced anxiety-like behaviors in mice [84]. It is important to note that the mechanism of γ-oryzanol is much broader than fenofibrate, but both cholesterol therapies showed an influence on adolescent alcohol-induced neuroimmune signaling.

### 6.5. Classic Anti-Inflammatory Drugs

The development of new immune modulators as well as revisiting classic anti-inflammatory drugs may be promising in impacting adolescent alcohol use and its long-term effects. For example, indomethacin is a COX-2 inhibitor drug currently prescribed to alleviate joint pain and swelling inflammation due to arthritis. In separate studies, indomethacin prevented [16,85] and reversed [86] AIE-induced reductions in neurogenesis as well as increases in neurodegeneration. Importantly, these therapeutic interventions also coincided with reductions in inflammatory markers, such as HMGB-1 or NF-κB [16,85,86]. Moreover, indomethacin pretreatment in an acute adolescent ethanol exposure model blocked alcohol-induced memory impairment as well as electrophysiological differences [15]. Similar findings were also found for both a TLR-4 antagonist, TAK-242, and minocycline [15]. TAK-242 is currently under investigation in clinical trials, including one for alcohol-induced liver damage. Likewise, ibudilast has shown clinical efficacy in reducing craving and consumption [87,88]. However, in the preclinical setting, ibudilast did not appear to attenuate alcohol-induced neuroimmune responses in adolescence, but PDE4 inhibitors do appear to be more effective in adults [89]. Minocycline is an antibiotic not classically considered an anti-inflammatory agent that has been suggested to reduce inflammation through an unknown mechanism [90]. More specifically, minocycline has been shown to reduce proinflammatory cytokines, astrocyte activation, and microglial activation, including in vitro and in vivo adult studies of AUDs [91,92,93]. Considering their shared effects on behavioral and electrophysiological maladaptations after ethanol, it is probable that minocycline, TAK-242, and indomethacin all influence the system through an anti-inflammatory mechanism.

### 6.6. Exercise

Growing evidence has emphasized exercise as a natural protector against excessive adolescent alcohol consumption and its effect on the neuroimmune responses and deficits in neurogenesis in the adult hippocampus. Multiple studies have shown exercise as a plausible therapy to treat brain injuries [16,42,94]. A study using an AIE rat model examined the effects of hippocampal neurogenesis from chronic alcohol exposure in conjunction with voluntary exercise during and after ethanol exposure. Animals were allowed voluntary exercise from P24 to 80, which encompassed both alcohol exposure in adolescence as well as the abstinence period into adulthood. Voluntary exercise prevented reductions in neurogenic markers DCX and nestin in the adult hippocampus as well as ameliorating the alcohol-induced increases in TNFα and IκBα in the adult brain. Likewise, in a follow-up study, the same group showed that voluntary exercise is a viable reversal strategy as the exercise was available only after AIE [94]. Furthermore, they showed a behavioral phenotype, as exercise also reversed AIE-induced learning and memory deficits in the Morris water maze [94]. A separate group’s research supported the potential of exercise to reverse AIE-induced neuroimmune dysregulation through the amelioration of cytokines and microglia in the hippocampus [42]. Exercise as a therapy for the restoration of persistent AIE-induced phenotypic dysregulations may be an intervention that could be coupled with pharmacologic therapies.

## 7. Conclusions

Adolescence represents a sensitive period of development when the brain is more malleable and can retain more impactful maladaptations. Unfortunately, this time of development is also associated with an increased prevalence of excessive ethanol consumption and binge drinking. Alcohol’s influence on the neuroimmune system in adolescents has lingering effects on the neural microenvironment and can change both behavioral and biological outcomes that contribute to the development and perpetuation of AUDs. In this analysis of the most recent findings of alcohol’s effects on the adolescent neuroimmune response, three key factors emerged in comparing the findings across laboratories and models that should influence future studies: (1) *Sex as a biological variable is critical in understanding the neuroimmune response after adolescent alcohol exposure*. There are so many variables related to sex that have the potential to interact with the effects of adolescent alcohol consumption on the neuroimmune response, including, but not limited to, the onset and end of adolescent brain development, alcohol metabolic differences [95,96], and especially the basal differences in glia and other neuroimmune factors by sex. Future studies should be powered to fully explore the role of sex in preclinical studies of alcohol-induced neuroimmune maladaptations to understand the potential differences that have been suggested in clinical studies. (2) *The phenomenon of neuroimmune dysregulation after adolescent alcohol misuse seems to be fairly universal, but the duration of ethanol consumption (acute vs. chronic) can change the players involved (e.g., cell type, brain regions, etc.)*. The findings from chronic, heavy alcohol use studies in adolescence seem to be more congruent with each other, even when there are differences in the doses compared to the acute studies. This is particularly troubling in models such as the DID where the neuroimmune response appears to be driven by more “moderate” binge alcohol levels rather than caused by alcohol-induced neurodegeneration. (3) *Immediate changes in the neuroimmune system during adolescent intoxication may persist but are often distinct from those observed in adulthood*. The persistent maladaptations in “reprogrammed” glial cells can exacerbate neuropathology as well as behavior, including repeated alcohol consumption after abstinence [97,98]. Considering the persistence of these neuroimmune responses, future studies should consider whether adolescent alcohol exposure after more prolonged abstinence into late adulthood is detrimental to the aging process, similar to studies in adults showing alcohol may exacerbate and/or mimic Alzheimer’s pathology [99,100,101]. In fact, recent work has shown that ethanol in adolescence exacerbates the pregenetic disposition for Alzheimer’s progression [102], but the propensity for spontaneous development has not been as readily studied. Despite the persistence of alcohol’s effects long after adolescence, there remains hope as neuroimmunomodulators have been shown to be able to ameliorate the long-term effects of adolescent alcohol use. The ability to reverse and prevent the long-term effects of alcohol on the neuroimmune response represents a great avenue for further study that has the potential to correct for both the behavioral and biological effects associated with alcohol-induced maladaptations.

## Figures and Tables

**Table 1:** ↑ Indicates an increase; -- indicates no change in comparisons to the control. ^†^ indicates dose response. * Indicates male-only effect. Toll-like receptor 1 (TLR1); toll-like receptor 2 (TLR2); toll-like receptor 3 (TLR3); toll-like receptor 4 (TLR4); toll-like receptor 5 (TLR5); toll-like receptor 6 (TLR6); toll-like receptor 7 (TLR7); toll-like receptor 8 (TLR8); toll-like receptor 9 (TLR9). PND = postnatal day.

Duration	Administration	Species/Strain	Toll-like Receptor Effects	Brain Region
***Acute***				
PND 22–25	Intragastric gavage (0.5 g/kg–3.5 g/kg/day)	Male & Female Balb/c mice	↑ TLR4 ^†^	Nucleus Accumbens
***Chronic***				
PND 28–42	Operant self-administration (~3.3 g/kg/day)	Male & Female Wistar Rats	↑ TLR4 *	Medial prefrontal cortex & Hippocampus
PND 25–55 [16]	Intragastric gavage (5 g/kg/day)	Male Wistar Rats	↑ TLR1, ↑ TLR2, -- TLR3, ↑ TLR4, ↑ TLR5, ↑ TLR6, ↑ TLR7, ↑ TLR8, -- TLR9	Hippocampus
PND ~30–51 [20]	Free access self-administration (~9.6 g/kg/day)	Male Wistar Rats	↑ TLR4	Hippocampus

**Table 2:** ↑ Indicates an increase; ↓ indicates a decrease; -- indicates no change in comparisons to the control. ^†^ Indicates dose response. * Indicates male-only effect. ** Indicates female-only response. Interleukin (IL); Tumor Necrosis Factor Alpha (TNF-α); C-C Motif Chemokine Ligand 2 (CCL2); C-C Motif Chemokine Ligand 11 (CCL11) Interferon Gamma (IFN-γ); Interleukin-4 (IL-4); Interleukin-10 (IL-10); C-X3-X Motif Ligand 1 (CX3CL1); High Mobility Group Box 1 (HMGB1); Cyclooxygenase-2 (COX2); Interferon Gamma-induced Protein 10 (IP-10); Lipopolysaccharide-induced CXC Chemokine (LIX); Regulated upon Activation Normal T-Cell Expressed and Presumable Secreted (RANTES); Transforming growth factor beta (TGF-β). PND = Postnatal day.

Duration	Administration	Species/Strain	Cytokine & Chemokine Effects		Brain Region
***Acute***			**Cytokines**	**Chemokines**	
PND 33–36	Intragastric gavage (~12 g/kg/day)	Male Sprague Dawley Rats	↓ IL-1β, ↓ IL-6, ↓ TNF-α, ↓ TGF-β	↓ CCL2	Hippocampus & Entorhinal Cortex
PND 32–33 [24]	Intraperitoneal (3.5–4 g/kg)	Male & Female Sprague Dawley Rats	↑IL-6	-	Hippocampus & Amygdala
PND 30–32	Intragastric gavage (3 or 6 g/kg/day)	Male Wistar Rats	↑↓ IFN-γ ^†^, -- TNF-α, IL-4, or IL-10	-	Prefrontal Cortex & Hippocampus
***Chronic***					
PND 29–41	Intraperitoneal (1.25 g/kg)	Male Oncins France-1 Mice	↑ IL-6	↑ CX3CL1	Striatum
~PND 30–56	Intragastric gavage (3 or 6 g/kg/day)	Male Wistar Rats	↑ IFN-γ, ↑ TNF-α, ↑ IL-4 (PFC), ↑ IL-10 (PFC)	-	Prefrontal Cortex & Hippocampus
PND 25–54	Intragastric gavage (5 g/kg/day)	Male Wistar Rats	-	↑ CCL2, ↑ HMGB1, ↑ COX2	Hippocampus
PND 30–52	Intragastric gavage (5 g/kg/day)	Male & Female Sprague Dawley Rats	-- TGF-β1, or IL-10, ↑ TNF-α *, ↑ IL-1β *	-	Hippocampus
PND 25–55 [16]	Intragastric gavage (5 g/kg/day)	Male Wistar Rats	↑ TNF-α, -- IL-4, TGF-β, or IL-10	↑ HMGB1	Whole Brain
PND 28–48 [31]	Intraperitoneal (4 g/kg/day)	Male & Female Sprague Dawley Rats	↓ TNF-α, ↑ IL-6, ↑ IL-1β ** (Hipp), ↓ IL-1β * (Amy)	-	Hippocampus & Amygdala
PND ~30–51 [20]	Free access self-administration (~9.6 g/kg/day)	Male Wistar Rats	↑ IL-1β, ↑ TNF-α, -- IL-6	-	Hippocampus
PND 25–38	Intraperitoneal (3.0 g/kg)	Male Sprague Dawley Rats	↑ IL-1β, ↑ TNF-α, ↑ IL-6	-	Hippocampus
PND 22–58	Ethanol Vapor (14 h/day)	Male Wistar Rats	↑ IL-1β, -- IL-4, IL-5, IL-13, IL-1α, IL12, IL-17, IL-18, IL-10, or TNF-α	↑ IP-10, -- CX3CL1, CCL11, LIX, or RANTES	Frontal Cortex

**Table 3:** ↑ Indicates an increase; ↓ indicates a decrease; -- indicates no change in comparisons to the control. * Indicates male only effect. ** Indicates female only response. ^#^ Sex or consumption details not indicated in study. PND = Postnatal day; * PND start for mice between 42 and 56, with the DID lasting approximately 18 days; Glial fibrillary acidic protein (GFAP); Ionized calcium-binding adaptor molecule 1 (IBA1); Dentate Gyrus (DG); Cornu Ammonis 1 (CA1); cluster of differentiation molecule 11b (CD11b); cluster of differentiation molecule 68 (CD68); cluster of differentiation molecule 45 (CD45); cluster of differentiation molecule 86 (CD86); cluster of differentiation molecule 32 (CD32); cluster of differentiation molecule 206 (CD206); Major histocompatibility complex II (MHC-II); Toll-like receptor 4 (TLR4); Cannabinoid receptor 1 (CB_1_); Connexin 43 (Cx43); Pannexin 1 (Panx1).

Duration	Administration	Species/Strain	Glial Effects		Brain Region
***Acute***			Astrocytes	Microglia	
PND 35–39	Intragastric gavage (3 g/kg/day)	Female Wistar Rats	↑ GFAP	-	Hippocampus
~PND 35–39	Intragastric gavage (5 g/kg/day)	Sprague Dawley Rats ^#^	-- GFAP	↑ Iba1	Hippocampus
PND 35–38 [39]	Intragastric gavage (5 g/kg)	Male Sprague-Dawley rats	-	↓ Iba1, ↑ Dystrophic microglia	Hippocampus, Perirhinal & Entorhinal Cortices
PND 33–36	Intragastric gavage (5 g/kg)	Male Sprague-Dawley rats	-	↑ CD11b, ↑ CD45, ↑ MHC-II, ↑ CD86 ↑ CD32, ↑ CD206	Hippocampus and Entorhinal Cortex
PND 40–42	Intraperitoneal (3 g/kg)	Male Sprague-Dawley rats	-- GFAP	-- Iba1, -- Microglia TLR4 expression	Hippocampus
***Chronic***					
~PND 42–74	DID self-administration (~3.5 g/kg/day)	Male & Female C57BL/6 N mice	-	↑ Iba1	Hippocampus
~PND 28–46	DID self-administration ^#^	Male C57BL/6 Mice	↑ GFAP	-	Hippocampus
PND 31–46	Intragastric gavage (5 g/kg/day)	Male & Female Sprague Dawley Rats	↑ GFAP	↑ Iba1 **	Hippocampus
~PND 30–58	Intragastric gavage (1–2 g/kg/day)	Male Sprague Dawley Rats	↑ GFAP (Hipp/HT), -- GFAP (PFC)	-	Hippocampus, Hypothalamus, Prefrontal Cortex
PND 24–55	Free access self-administration ^#^	Male Syrian Mice	↑ GFAP	-	Hippocampus
PND 28–50	Intragastric gavage (5 g/kg)	Male Sprague Dawley Rats	↓ astrocytic-synaptic contact, -- GLT-1, GLAST, α2-δ1, or PSD-95	-	Medial prefrontal cortex
PND 30–46	Intragastric gavage (5 g/kg)	Male Sprague Dawley Rats	*During Withdrawal*-- Astrocyte volume or synaptic contact	-	Medial prefrontal cortex, Orbitofrontal cortex, Anterior cingulate cortex
			*Persisting Effects*-- Astrocyte Volume ↓ astrocytic-synaptic contact (ACC/OFC)		
~PND 32–84 ^(start & duration varied)^	DID self-administration (~2.5 g/kg/day)	Male C57BL/6 Mice	↓ Astrocytic CB_1_ and number of processes ↑ Area	-	Hippocampus
PND 31–46	Intragastric gavage (5 g/kg)	Male Sprague Dawley Rats	↓ Astrocytic-synaptic proximity	-	Hippocampus
PND 25–38	Intraperitoneal (3 g/kg)	Male Sprague Dawley Rats	↑ Cx43 and Panx1 activity ↑ Arborization	-	Hippocampus
PND 35 -58	Intragastric gavage (3 g/kg/day)	Female Wistar Rats	↑ GFAP	-	Hippocampus
PND 30–46	Intragastric gavage (5 g/kg)	Male & Female Sprague Dawley Rats	↑ GLT-1 ↑ xCT and GLAST *	-	Hippocampus
PND 28–55	Intragastric gavage (5 g/kg)	Male Wistar Rats	-	↑ Iba1 and CD68	Hippocampus
PND 28–49	Free access self-administration (~9.6 g/kg/day)	Male Wistar Rats	-	↑ Iba1	Hippocampus
~PND 25–53	Free access self-administration (~13.5 g/kg/day)	Male C57BL/6 Mice	-	↑ CD68	Dorsal horn, amygdala, anterior cingulate cortex, medullary raphe, thalamus, & hypothalamus
PND 28–43	Intragastric gavage (3.5 g/kg)	Male C57BL/6 Mice	-	↓ Iba1	Hippocampus
PND 28–42	Operant self-administration (~3.3 g/kg/day)	Male & Female Wistar Rats	-	↑ Ibal, ↓ Ramified **	Hippocampus & prefrontal cortex
PND 22–58	Ethanol Vapor (14 h/day)	Male Wistar Rats	-	↑ Iba1	Amygdala, frontal cortex, hippocampus, and substantia nigra

## Data Availability

No new data were created or analyzed in this study. Data sharing is not applicable to this article.
